# Impact of supplementation with a food-derived microbial community on obesity-associated inflammation and gut microbiota composition

**DOI:** 10.1186/s12263-017-0583-1

**Published:** 2017-10-04

**Authors:** Marianna Roselli, Chiara Devirgiliis, Paola Zinno, Barbara Guantario, Alberto Finamore, Rita Rami, Giuditta Perozzi

**Affiliations:** Food and Nutrition Research Centre, Council for Agricultural Research and Economics (CREA), Via Ardeatina 546, 00178 Rome, Italy

**Keywords:** Chronic inflammation, Fermented dairy, Foodborne microbiota, White adipose tissue, High fat diet

## Abstract

**Background:**

Obesity is a complex pathology associated with dysbiosis, metabolic alterations, and low-grade chronic inflammation promoted by immune cells, infiltrating and populating the adipose tissue. Probiotic supplementation was suggested to be capable of counteracting obesity-associated immune and microbial alterations, based on its proven immunomodulatory activity and positive effect on gut microbial balance. Traditional fermented foods represent a natural source of live microbes, including environmental strains with probiotic features, which could transiently colonise the gut. The aim of our work was to evaluate the impact of supplementation with a complex foodborne bacterial consortium on obesity-associated inflammation and gut microbiota composition in a mouse model.

**Methods:**

C57BL/6J mice fed a 45% high fat diet (HFD) for 90 days were supplemented with a mixture of foodborne lactic acid bacteria derived from the traditional fermented dairy product “Mozzarella di Bufala Campana” (MBC) or with the commercial probiotic GG strain of *Lactobacillus rhamnosus* (LGG). Inflammation was assessed in epididymal white adipose tissue (WAT) following HFD. Faecal microbiota composition was studied by next-generation sequencing.

**Results:**

Significant reduction of epididymal WAT weight was observed in MBC-treated, as compared to LGG and control, animals. Serum metabolic profiling showed correspondingly reduced levels of triglycerides and higher levels of HDL cholesterol, as well as a trend toward reduction of LDL-cholesterol levels. Analysis of the principal leucocyte subpopulations in epididymal WAT revealed increased regulatory T cells and CD4^+^ cells in MBC microbiota-supplemented mice, as well as decreased macrophage and CD8^+^ cell numbers, suggesting anti-inflammatory effects. These results were associated with lower levels of pro-inflammatory cytokines and chemokines in WAT explants. Faecal bacterial profiling demonstrated increased *Firmicutes*/*Bacteroidetes* ratio in all mice groups following HFD.

**Conclusions:**

Taken together, these results indicate a protective effect of MBC microbiota supplementation toward HFD-induced fat accumulation and triglyceride and cholesterol levels, as well as inflammation, suggesting a stronger effect of a mixed microbial consortium vs single-strain probiotic supplementation. The immunomodulatory activity exerted by the MBC microbiota could be due to synergistic interactions within the microbial consortium, highlighting the important role of dietary microbes with yet uncharacterised probiotic effect.

**Electronic supplementary material:**

The online version of this article (10.1186/s12263-017-0583-1) contains supplementary material, which is available to authorized users.

## Background

Obesity is a chronic, multifactorial disorder reaching epidemic proportions globally, affecting persons of virtually all ages in both developed and developing countries [1, 2]. Promoted by a combination of genetic predisposition, nutritional excess, and sedentary lifestyle, obesity is primarily characterised by increased fat mass, accompanied by development of related disorders [3–5]. Expansion of the adipose organ, mainly affecting white adipose tissue (WAT), results in adipocyte dysfunction. WAT has been increasingly considered not only a metabolic organ, but also an active endocrine tissue, as it secretes a large number of peptide hormones called adipokines, such as leptin and adiponectin, that operate in a complex network and actively communicate with other organs [6, 7]. Secretion by the adipose organ is disturbed in obesity, as adipokine release is dysregulated and associated with production of several inflammation mediators. For this reason, the adipose tissue is considered to be a major contributor to obesity-linked low-grade chronic inflammation [8]. The inflammatory process involves impairment of both the innate and adaptive immune system and is triggered by local secretion of inflammatory cytokines and chemokines such as tumour necrosis factor-α (TNF-α), interleukin-6 (IL-6), monocyte chemoattractant protein (MCP)-1, and Regulated on Activation Normal T cell Expressed and Secreted (RANTES). These mediators recruit immune cells from blood vessels, such as lymphocytes and macrophages, which in turn massively infiltrate the adipose tissue [9]. Indeed, high levels of inflammatory cells such as T CD8^+^ lymphocytes and activated M1 macrophages are found in obese WAT, accompanied by decreased levels of CD4^+^CD25^+^Foxp3^+^ regulatory T (Treg) cells, a key population in maintaining immunological tolerance and immune homeostasis [10–12]. This inflammatory status, arising locally and then becoming systemic, triggers the onset of other diseases frequently associated with obesity such as the metabolic syndrome, characterised by visceral obesity, high blood pressure, insulin resistance, high circulating triglyceride levels, and low HDL cholesterol, leading in turn to increased risk of cardiovascular diseases [13–16].

The gut microbiota has recently attracted much attention as a crucial factor associated with obesity [17]. Alterations of intestinal microbial composition, in terms of bacterial phyla and classes associated with improved energy extraction from the undigested dietary carbohydrate component, were identified in obese human subjects and animal obesity models, with consequent impact on host metabolism and energy storage [18]. Both diet- and genetically induced obesity were shown to associate with imbalance in the relative proportion of Gram-negative *Bacteroidetes* and Gram-positive *Firmicutes*, the two major phyla of gut bacteria, with the latter prevailing in obese subjects [19]. However, imbalance in these two bacterial phyla is not sufficient by itself to determine the obesity phenotype. Other factors, such as diet, pre- and probiotic supplementation, antibiotics, surgery, and faecal transplantation, can impact the overall metabolic capacity of the gut microbiome [20]. Within this context, dietary interventions aimed at promoting selection of beneficial intestinal microbes could represent a powerful strategy to counteract obesity-associated intestinal dysbiosis. There is growing evidence that probiotic and/or prebiotic supplementation can positively modulate gut microbiota, thus representing important assets in the management of obesity [21]. The probiotic component of the gut microbiota can confer health benefits to the host mainly acting on immunomodulation and positively influencing intestinal microbial balance [22]. Probiotic supplementation was therefore suggested to be able to counteract obesity-associated immune alterations and microbial imbalance [23–25]. As an alternative to commercially available probiotic strains, a natural source of live bacteria is represented by fermented foods, which also confer the advantage of providing the host with a complex microbiota containing several environmental strains with potential probiotic features, such as the capability to transiently colonise animal and human gut and interact with the resident gut microbiota, mainly at a trophic level [26]. Increasing scientific interest in fermented foods was also recently boosted by their possible use as models for more complex microbiota such as the gut [27]. The most relevant foodborne lactic acid bacteria (LAB) belong to the *Lactobacillus*, *Lactococcus*, *Streptococcus*, *Pediococcus*, and *Leuconostoc* genera. Several LAB species are also highly represented within the resident gut microbiota of healthy humans. *Lactobacillus* species, in particular, are abundant both in food and in the gut [28].

The aim of our work was to evaluate the impact of supplementation with a complex foodborne bacterial community on obesity-associated inflammation, as well as on gut microbiota composition. For this purpose, we used a mouse model of high fat diet (HFD)-induced obesity, comparing the effect of supplementation with a mixture of natural LAB strains derived from the traditional fermented dairy product “Mozzarella di Bufala Campana” (MBC) [29] and with the well-characterised probiotic GG strain of *Lactobacillus rhamnosus* (LGG). The MBC bacterial consortium was dominated by *Lactobacillus delbrueckii*, *Lactobacillus fermentum*, and *Leuconostoc lactis* [30]. LGG was used as probiotic control on the basis of its proven beneficial effects in the prevention of obesity [31, 32].

## Methods

### Experimental design, animals, and diets

**Fig. 1:**
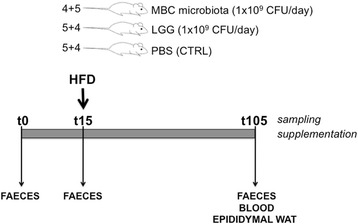
Experimental design. Six-week-old C57BL/6J male mice were randomly assigned to three experimental groups (four or five animals per group). The mice were fed a standard diet and orally supplemented daily with MBC microbiota, LGG, or PBS (CTRL). After 15 days, the mice were shifted to HFD while continuing bacterial or PBS supplementation for 90 additional days. At the end of the experimental period, blood and epididymal WAT were collected. Faeces were sampled for gut microbiota analysis at the indicated time points: t0, t15, and t105. The experiment was replicated once, and the number of mice in each group for each of the two repetitions is indicated

Six-week-old C57BL/6J male mice, obtained from Charles River Laboratories (Como, Italy), were kept at 23 °C with a 12-h light-dark cycle and fed ad libitum with a standard laboratory diet (4RF21, Mucedola, Milano, Italy, www.mucedola.it). Mice had free access to food and water throughout the experiments. Food intake and body weight were recorded every other day. After 1 week of adaptation, animals were randomly divided into three groups (five mice per group) and orally supplemented for 15 days with 1 × 10^9^ CFU/day of a mixture of natural LAB strains extracted from MBC [29] or with the probiotic strain LGG. Phosphate-buffered saline (PBS) supplementation was used as control (CTRL). After 15 days, all mice were shifted to HFD (http://www.envigo.com/resources/data-sheets/06415.pdf, 44.8% total calories from fat, designed with similarities to Research Diets, Inc., formula D12451 and provided by Mucedola) while continuing to receive bacterial supplementation for 90 additional days. Due to logistic reasons related to the number of animals that could be handled at the same time, the experimental design envisioned two rounds of treatment, 2 weeks apart from each other, in which the two groups of mice, of the same age, were fed the same batches of diets. Therefore, the second group of mice was not aimed at testing reproducibility, but rather at increasing the number of treated animals. Statistical analysis of the results included all animals subjected to the same supplementation protocol, irrespective of their treatment within experimental period 1 or 2. At the end of the experimental period, mice were anaesthetised by intraperitoneal injection of pentobarbital (10 mg/kg) following overnight fasting, blood was drawn via cardiac puncture, and epididymal WAT was excised, weighed, and immediately placed in ice-cold PBS under sterile conditions. Serum was prepared from blood and stored at − 80 °C until further analysis. Faeces were collected and stored at − 80 °C for microbiological analysis at the following time points: t0 (beginning of bacterial treatments), t15 (shift to HFD) and t105 (90 days on HFD). The experimental protocol and sampling times are summarised in Fig. 1.

### Bacterial preparations

MBC is a traditional Italian fermented cheese with PDO designation (Product of Designated Origin, EEC Regulation no. 1107). It is consumed fresh, within 2 weeks from production, and it contains high titres of live bacteria [29]. To prepare the MBC microbiota, 10 g of cheese samples were diluted in 90 ml sodium citrate solution (2% *w*/*v*) and homogenised in a BagMixer400 (Interscience, France), as previously described [30]. To standardise the bacterial inoculum to be administered to mice, the MBC homogenate was entirely used as a single inoculum in 2 l of De Man Rogosa Sharpe (MRS) medium (Oxoid Ltd., England) and incubated at 37 °C for 48 h under anaerobic conditions (Anaerocult A, Merck, Germany) to obtain a final bacterial titre of about 1.5 × 10^9^ CFU/ml. The resulting bacterial suspension was divided in aliquots containing 1 × 10^9^ CFU each, stored at − 80 °C in 20% (*v*/*v*) glycerol, and thawed daily for oral administration to mice, following washing, resuspension in 1× PBS, and mixing with small amounts of minced feed.

The LGG strain ATCC53103 was grown, prepared, and orally given to mice as described above for MBC microbiota.

### Serum metabolic measurements

The following plasma parameters were analysed: glucose (Glucose Liquid kit, Sentinel Diagnostics, Milan, Italy), HDL and LDL cholesterol (Max Discovery HDL and LDL Cholesterol Assay Kits, Bioo Scientific, Austin, TX), and triglycerides (Triglycerides Liquid kit, Sentinel Diagnostics). The adiponectin was quantified by ELISA (Biorbyt, Cambridge, UK). Analyses were conducted on a subset of five samples for each treatment, due to technical issues related to serum withdrawal or hemolysis.

### Immune cell isolation and staining

Macrophages and lymphocytes were isolated from the epididymal WAT stromal vascular fraction (SVF), according to [33], as several populations of immune cells are well known to reside in the SVF. The following monoclonal antibodies, purchased from eBioscience (San Diego, CA), were used in this study: FITC anti-CD3 (clone 500A2), PE anti-CD8 (clone 53-6.7), PE-Cy5 anti-CD4 (clone RM4-5), FITC anti-CD11b (clone M1/70), PE anti-F4/80 (clone BM8), PerCP-Cy5.5 anti-CD45 (clone 30-F11), and anti-CD16/CD32 (clone 93). Briefly, 1 × 10^6^ cells, resuspended in FACS labelling buffer (PBS with 2 mM EDTA and 1% foetal calf serum), were pre-incubated for 20 min with anti-CD16/CD32 to avoid non-specific binding, then washed and labelled with the appropriate mixture of antibodies for 30 min, centrifuged, and resuspended in FACS labelling buffer. Flow cytometry analysis was performed using a FACSCalibur flow cytometer (BD Biosciences, Milan, Italy). To exclude dead/dying cells that could non-specifically bind antibodies, leukocytes were gated according to forward and side scatter. The percentage of T helper and cytotoxic cells was calculated on lymphocyte gate (CD3^+^), whereas the CD11b^+^ and F4/80^+^ cell subsets were calculated on the leukocyte gate (CD45^+^). Treg cell (CD4^+^CD25^+^Foxp3^+^) analysis was performed with a specific kit (eBioscience, San Diego, CA) staining CD4 (FITC), CD25 (PE) and transcription factor Foxp3 (PE-Cy5), according to the manufacturer’s instructions. The percentage of CD25^+^Foxp3^+^ cells was calculated on lymphocyte CD4^+^ gate. For all analyses, at least 10.000 events were acquired and analysed using the CellQuest software (BD Biosciences, Milan, Italy).

### Cytokine and chemokine secretion in WAT explants

WAT explant cultures were established essentially as described by [34]. Briefly, epididymal WAT was dissected, weighed, minced, and placed into 12-well tissue culture plates (Corning, Milan, Italy) at 120 mg/well, with either 1 ml T cell activation medium (complete DMEM containing 3.7 g/l NaHCO_3,_ 10% heat-inactivated foetal calf serum, 4 mM glutamine, 1% non-essential amino acids, 10^5^ U/l penicillin and 100 mg/l streptomycin, 5 ng/ml phorbol 12-myristate 13-acetate (PMA), and 1 ng/ml ionomycin) or control medium (complete DMEM without ionomycin and PMA). All reagents were from Euroclone (Milan, Italy), except for ionomycin and PMA, which were from Sigma (Milan, Italy). Conditioned media were collected after 24 h of culture at 37 °C in an atmosphere of 5% CO_2_/95% air at 90% relative humidity and stored at − 80 °C until further analysis. The levels of cytokines and chemokines were analysed using Bio-plex/Luminex technology (mouse magnetic Luminex screening assay, Labospace, Milan) or ELISA assays (Affymetrix, eBioscience, San Diego, CA). The following cytokines and chemokines were simultaneously detected by Luminex technology in 50 μl undiluted samples: interferon gamma-induced protein (IP)-10, granulocyte macrophage-colony stimulating factor (GM-CSF), Regulated on Activation-Normal T cell Expressed and Secreted (RANTES), interleukin (IL)-23, IL-4, and IL-10. The following cytokines were analysed by ELISA (100 μl samples): tumour necrosis factor (TNF)-α, interferon (IFN)-γ, IL-17A, and IL-6. For these latter two cytokines, samples were diluted 1:500, as the readings by Luminex assays for IL-17A and IL-6 were out of range.

### DNA extraction from faecal samples

Total DNA was extracted from 80 mg faecal samples with QIAamp DNA Stool Mini Kit (Qiagen, Hilden, Germany) according to manufacturer’s instructions. Qiagen DNA extraction method used in this work was chosen as it was listed among the most reproducible kits, ensuring minimal influence on next-generation sequencing (NGS) data analysis [35].

### NGS analysis

NGS was performed on faecal DNA samples from four animals for each of the three experimental groups, at the three time points indicated in Fig. 1, namely t0, t15, and t105 (total number of samples = 36). Partial 16S rRNA gene sequences were amplified using primer pair Probio_Uni and /Probio_Rev, which targets the V3 region of the gene and sequenced at the DNA sequencing facility of GenProbio srl (www.genprobio.com) using a MiSeq (Illumina). Primers and protocols, including amplicon checks, were as described in [36]. Individual sequence reads were filtered with the Illumina software to remove low quality and polyclonal sequences. All Illumina quality-approved, trimmed, and filtered data were exported as .fastq files and processed using a custom script based on the QIIME software suite [37]. Quality control retained sequences 140–400 bp long, with mean sequence quality score > 20, and truncation at first base if a low quality rolling 10-bp window was found. Presence of homopolymers > 7 bp and sequences with mismatched primers were omitted. To calculate downstream diversity (alpha and beta diversity indices, UniFrac analysis), 16S rRNA operational taxonomic units (OTUs) were defined at ≥ 97% sequence homology using uclust [38]. All reads were classified to the lowest possible taxonomic rank using QIIME and a reference dataset from the SILVA database [39]. Similarities between samples were calculated by unweighted UniFrac [40]. The range of similarities is calculated between the values 0 and 1. Principal Coordinate Analysis (PCoA) was applied using the UniFrac program.

### Statistical univariate analysis

Values in graphs and tables represent means ± SD. Prior to analysis, normal distribution and homogeneity of variance of all variables were assumed with Shapiro-Wilk’s and Levene’s tests, respectively. Statistical significance was evaluated by one-way ANOVA or by ANCOVA, followed by post hoc Tukey honestly significant difference (HSD) test. Differences with *P* values < 0.05 were considered significant. Statistical univariate analysis was performed with the “Statistica” software package (version 5.0; Stat Soft Inc., Tulsa, OK).

### Statistical multivariate analysis

Non-supervised principal component analysis (PCA) of WAT immunological profiles (leukocyte subpopulations and cytokine/chemokine secretion) was performed with Past software, version 2.17c [41]. Data were collected in a matrix of 27 rows (number of animals) and 15 columns (number of variables) and were auto-scaled by mean-centring and normalised by standard deviation. Pearson’s correlation coefficients between variables and principal components, as well as statistical significance of the correlation, were also calculated.

## Results

### Bacterial supplementation affects epididymal WAT weight and metabolic parameters

Body and WAT weight values in the three groups of mice are shown in Table 1 in comparison with food and energy intakes. As expected, HFD feeding induced significant weight increase in all groups, leading to comparable body weight and weight gain values by the end of the experimental period. Nevertheless, significant reduction of WAT weight (*P* < 0.05) was observed in MBC-treated animals, as compared to LGG and CTRL mice. Food and energy intake were similar in the three mice groups. To account for a possible influence of food intake on WAT weight, ANCOVA analysis was performed, considering WAT weight as the dependent variable, treatment as the independent variable, and food intake as the covariate. The results confirmed that WAT weight reduction in the MBC group as compared to LGG and CTRL could not be attributable to differential food intake. Supplementing with the foodborne MBC microbiota also led to reduced serum levels of triglycerides, coupled with higher levels of HDL cholesterol (*P* < 0.05 and *P* < 0.001, respectively), and a trend toward decreased LDL cholesterol (*P* = 0.05) as compared to the CTRL group (Table 2). Serum metabolic parameters of LGG-treated mice displayed a similar but milder effect, with a trend toward reduced triglyceride levels (*P* = 0.05) and increased HDL-cholesterol levels (*P* < 0.05). No significant differences were detected among the three groups of mice concerning fasting glucose and adiponectin levels.

**Table 1:** Body weight, epididymal WAT weight, and food and energy intake from HFD of MBC, LGG, or CTRL mice

	MBC	LGG	CTRL
Body weight (g)			
Initial	19.19 ± 1.44	19.41 ± 1.79	18.80 ± 0.89
Final	31.75 ± 1.97	31.88 ± 2.13	30.63 ± 3.35
Gain	11.61 ± 1.94	12.47 ± 2.55	12.36 ± 2.63
WAT weight (g)	1.35*^#^ ± 0.31	1.78 ± 0.30	1.89 ± 0.49
Food intake (g/day)	2.40 ± 0.66	2.54 ± 0.62	2.48 ± 0.63
Energy intake from HFD (Kcal/day)	10.80 ± 0.60	11.39 ± 0.69	11.26 ± 1.05

*P < 0.05 versus CTRL (ANOVA); ^#^ *P* < 0.01 versus CTRL and LGG (ANCOVA)

**Table 2:** Serum metabolic measurements in MBC, LGG, or CTRL mice

	MBC	LGG	CTRL
Glucose (mg/dl)	107.42 ± 47.74	72.97 ± 7.64	86.91 ± 11.43
Triglycerides (mg/dl)	147.12* ± 65.91	163.21^#^ ± 69.23	316.98 ± 142.39
HDL cholesterol (mg/dl)	155.91** ± 16.13	137.29* ± 25.21	108.86 ± 12.91
LDL cholesterol (mg/dl)	92.75^#^ ± 19.06	105.77 ± 48.03	132.83 ± 15.09
Adiponectin (μg/ml)	16.25 ± 3.39	17.46 ± 4.87	19.23 ± 5.53

*P < 0.05 versus CTRL; **P < 0.001 versus CTRL; ^#^ *P* = 0.05 versus CTRL

### WAT immunological profiles highlight the anti-inflammatory effect of MBC microbiota supplementation

**Fig. 2:**
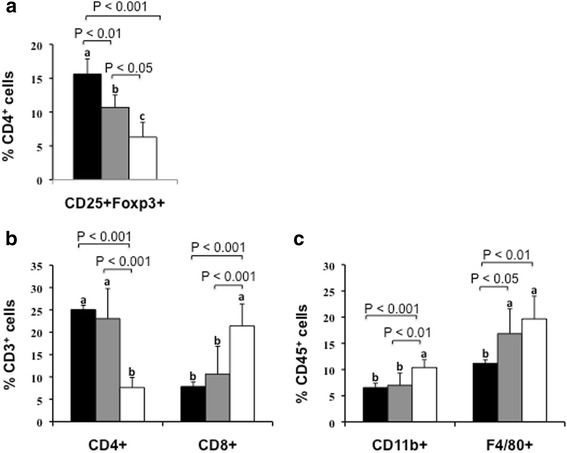
Leukocyte subpopulations in epididymal WAT. The effect of bacterial supplementation on the frequency of WAT leukocyte subpopulations was analysed by flow cytometry. The percentage of CD25^+^Foxp3^+^ Treg cells was calculated on T lymphocyte gate (CD4+, **a**), CD4^+^ and CD8^+^ cell subsets were calculated on lymphocyte gate (CD3^+^, **b**), whereas CD11b^+^ and F4/80^+^ cells were calculated on leukocyte gate (CD45^+^, **c**). Black columns: MBC-supplemented mice; grey columns: LGG-supplemented; white columns: CTRL. Each column represents the mean ± SD of nine mice. Means without a common letter significantly differ

Flow cytometry analysis of the main leukocyte subpopulations in epididymal WAT (Fig. 2) revealed increased numbers of the immune homeostasis regulator CD4^+^ CD25^+^ Foxp3^+^ Treg cells (Fig. 2a, *P* < 0.001 vs CTRL and *P* < 0.01 vs LGG) and CD4^+^ T lymphocytes (Fig. 2b, *P* < 0.001 vs CTRL) in MBC microbiota-supplemented mice, accompanied by decreased pro-inflammatory CD8^+^ T lymphocytes (Fig. 2b, *P* < 0.001 vs CTRL), CD11b^+^ activated leukocytes and F4/80^+^ macrophages (Fig. 2c, *P* < 0.001 and *P* < 0.01 vs CTRL, respectively), suggesting that MBC supplementation associates with an overall anti-inflammatory effect. LGG treatment also positively affected WAT leukocyte subpopulations in terms of increased percentage of Treg (*P* < 0.05 vs CTRL) and CD4^+^ cells (*P* < 0.001 vs CTRL) and decreased CD8^+^ cells (*P* < 0.001 vs CTRL) as well as activated leukocytes (*P* < 0.01 vs CTRL).

**Fig. 3:**
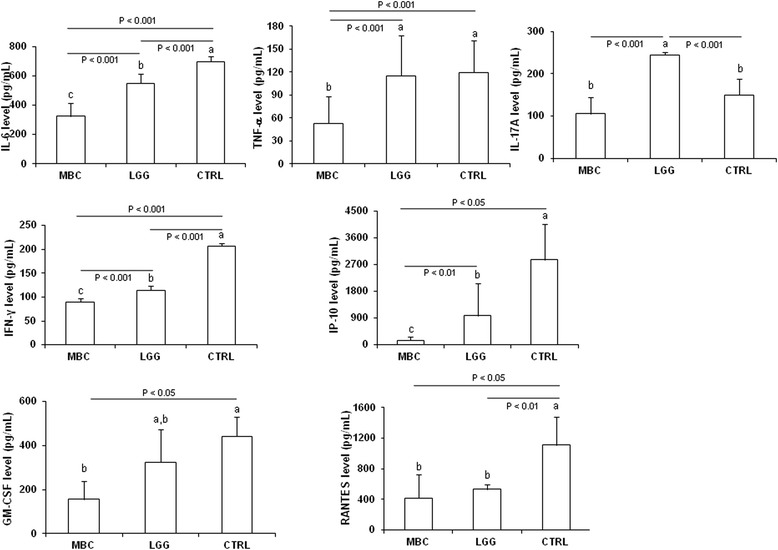
Cytokine and chemokine secretion in epididymal WAT explants. WAT explants were cultured in complete DMEM for 24 h in the presence of ionomycin (1 ng/ml) and PMA (5 ng/ml). Cytokine and chemokine levels were analysed by Luminex assay or by ELISA (see the “Methods” section). Each column represents the mean ± SD of nine mice. Means without a common letter significantly differ

Leukocyte profiling of MBC-treated animals was associated in cultured WAT explants with decreased levels of pro-inflammatory cytokines and chemokines, such as IL-6, TNF-α and IFN-γ (*P* < 0.001 vs CTRL and LGG), IL-17A (*P* < 0.001 vs LGG), IP-10 (*P* < 0.01 vs LGG and *P* < 0.05 vs CTRL), GM-CSF, and RANTES (*P* < 0.05 vs CTRL). Reduced levels were also observed in WAT leukocytes of LGG-supplemented mice, but they related to a smaller subset of pro-inflammatory cytokines, namely IL-6 and IFN-γ (*P* < 0.001 vs CTRL), IL-17A, and RANTES (*P* < 0.001 and *P* < 0.01 vs CTRL, respectively) (Fig. 3). No significant differences were observed among mice groups for the two anti-inflammatory cytokines IL-4 and IL-10 nor for pro-inflammatory IL-23 (data not shown).

**Fig. 4:**
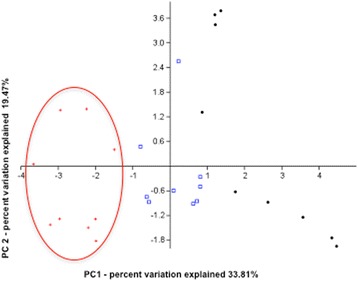
PCA plot from epididymal WAT immunological profiles. PC1/PC2 score plot showing the distribution of samples in reduced PC1/PC2 space. The percentage variation explained by the plotted principal components is indicated. Symbols refer to individual mice. Red crosses: MBC-supplemented mice; blue squares: LGG-supplemented; black dots: CTRL

Considering the dynamic and inherently multivariate nature of the immune response, WAT immunological profiles were further explored by principal component analysis (PCA) (Table 3). The first three principal components accounted for 64.15% of the overall variance, with individual values of 33.81, 19.47, and 10.87% for PC1, PC2, and PC3, respectively. The most informative score plot was the PC1/PC2 shown in Fig. 4, where PC1 was responsible for clearly discriminating MBC samples from LGG and CTRL samples. The variables mostly contributing to such discrimination are identified by higher loading values on PC1 (presented in italic characters in Table 3), indicating significant correlation between PC1 and the specific variable. In particular: PC1 shows strong significant inverse correlation with the pro-inflammatory markers CD3CD8^+^ (*r* = − 0.813), CD11b^+^ (*r* = − 0.727), F4/80^+^ (*r* = − 0.804), IL-6 (*r* = − 0.669), TNF-α (*r* = − 0.660), and GM-CSF (*r* = − 0.544) and significant direct correlation with the anti-inflammatory markers CD3CD4^+^ (*r* = 0.778) and CD4CD25^+^ (*r* = 0.819). However, a tendency of the LGG and CTRL samples to separate into two distinct clusters is also observed (Fig. 4). PC2, on the other hand, discriminates a subgroup of CTRL mice showing both pro- and anti-inflammatory features. These features are highlighted by the most discriminative variables: the pro-inflammatory cytokines IP-10 (*r* = 0.842) and IFN-γ (*r* = 0.587) and the anti-inflammatory markers IL-4 (*r* = 0.733) and IL-10 (*r* = 0.763) (Table 3).

**Table 3:** PCA loadings relative to the first two principal components from WAT immunological profiles of MBC, LGG, and CTRL mice

	PC1	PC2
CD8^+^	*0.3609*	−0.1459
CD4^+^	*−0.3455*	0.0893
CD4CD25^+^	*−0.3635*	0.0669
CD11b^+^	*0.3228*	−0.1137
F4/80^+^	*0.3569*	−0.0599
GM-CSF	*0.2416*	−0.1880
RANTES	0.1786	−0.1534
IL-23	0.1371	−0.1230
IP-10	0.1248	*0.4924*
IL-4	−0.0169	*0.4287*
IL-10	0.0720	*0.4465*
IL-6	*0.2973*	0.3429
IL-17A	0.1223	0.0648
IFN-γ	0.2593	*0.3435*
TNF-α	*0.2932*	−0.1015

Positive or negative values indicate a direct or inverse correlation between variables and PCs, respectively. Loading values associated to significant Pearson’s correlation coefficients (reported in the text) are indicated in italics

### Impact of bacterial supplementation on gut microbiota profiles

**Fig. 5:**
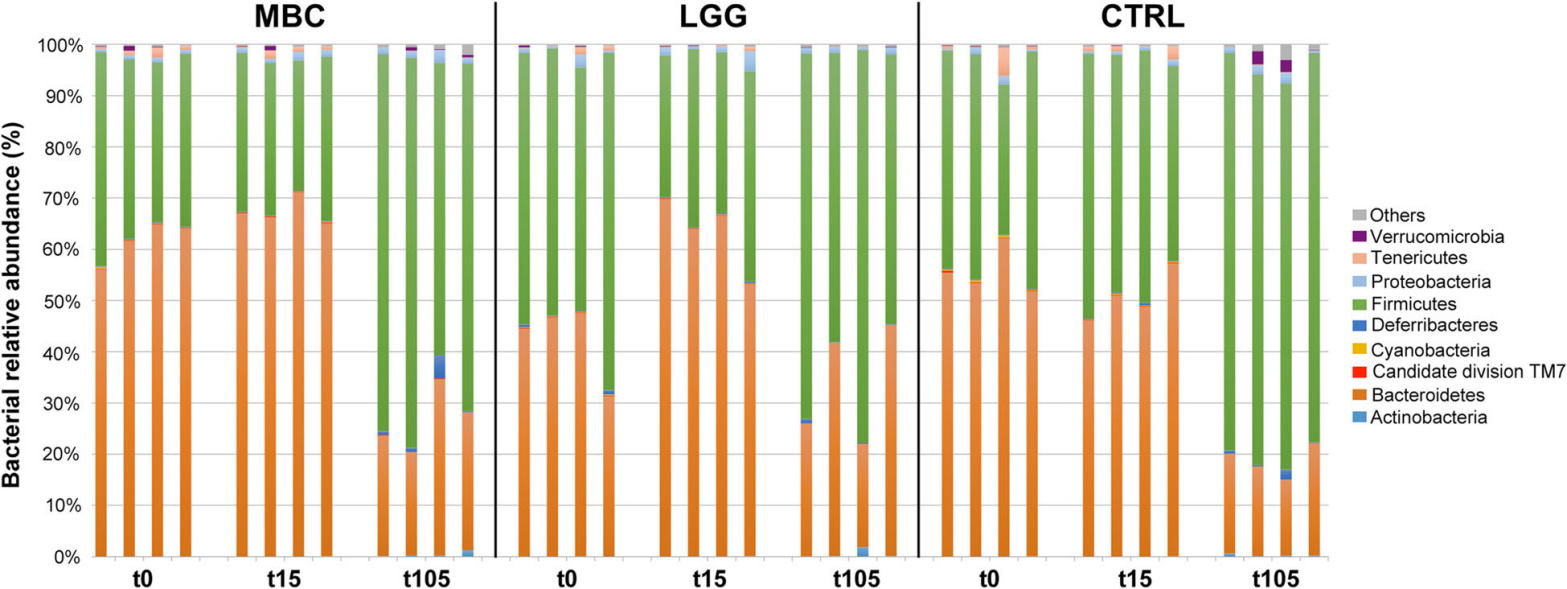
Relative abundance of gut bacterial *phyla* obtained by NGS of faecal samples. Each bar refers to a single faecal sample and depicts the proportion of OTUs per sample, expressed as percentage. Colour coding of bacterial *phyla* is shown on the right side. “Others” includes unidentified microorganisms of Bacteria kingdom or of Eukaryota kingdom and unclassified microorganisms

**Fig. 6:**
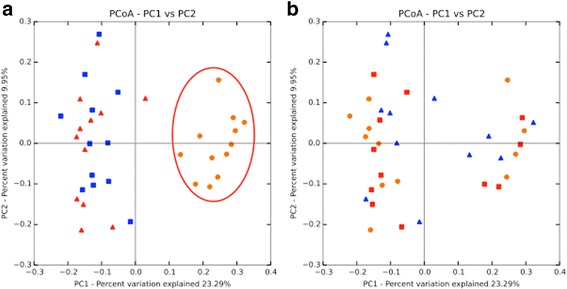
PCoA plots of unweighted UniFrac distance matrix. PC1/PC2 score plot showing the distribution of samples. The same plots are shown in each panel, with symbols referring to individual samples, but colour coding of each sample refers to time points in **a** (t0 = red triangles, t15 = blue squares, t105 = orange circles) or treatment type in **b** (CTRL = red triangles, LGG = blue squares, MBC = orange circles). The percentage variation explained by the plotted principal coordinates is indicated in the axis legend. Score values shown along the axes represent the proportion of dissimilarities captured by each axis

Next-generation sequencing (NGS) of 16S rDNA from faecal samples of treated or control mice was used to retrieve information on the bacterial relative abundance at time points t0, t15, and t105. Taxonomical assignment and read abundance estimates for all detected operational taxonomic units (OTUs) are reported in Fig. 5 at the *phylum* level, while the corresponding profiles at the species level are listed in Additional file 1: Table S1. As expected, *Bacteroidetes* and *Firmicutes* were detected as predominant bacterial *phyla*, with different relative proportions related to the time points analysed (Fig. 5). Notably, all three experimental groups displayed statistically significant increase in the *Firmicutes*/*Bacteroidetes* ratio at the final time point as compared to the beginning of the HFD treatment (t105 vs t15: *P* < 0.001 for MBC and LGG; *P* < 0.05 for CTRL). These altered ratios were also accompanied by decreased microbial biodiversity, measured by the Chao1 and Shannon indices (data not shown). Differences in the overall composition of the faecal bacterial community were further analysed using the UniFrac phylogeny-based metric [40]. Principal Coordinates Analysis (PCoA) confirmed clustering of bacterial species according to sampling time. The first three principal components accounted for 41% of the overall variance, with individual values of 23, 10, and 8% for PC1, PC2, and PC3, respectively. The most informative score plot was the PC1/PC2, shown in Fig. 6. A clear difference was observed between the initial (t0, t15) and final (t105) time points (Fig. 6a), while no difference could be observed among the three experimental conditions when samples were grouped according to supplementation type (Fig. 6b). However, it is worth noting that both *L*. *delbrueckii* and *Leuc*. *lactis* species, representing two major components of the MBC microbiota [29, 30], were detected exclusively in faecal samples of MBC-supplemented mice, although at very low abundance (Additional file 1: Table S1).

## Discussion

In this work, we investigated the effects of a complex foodborne bacterial community (MBC microbiota) on obesity-associated inflammation and gut microbiota composition in a HFD-induced obese mouse model. The cultivable LAB component of MBC microbiota, selected by growth in MRS medium, was extracted from a fermented unripened cheese especially rich in live titres of LAB species [29] dominated by *L*. *fermentum*, *L*. *delbrueckii*, and *Leuc*. *lactis* [30] whose strains have often been associated with probiotic features [42]. The rationale for supplementing mice with the microbial consortium was based on the highly biodiverse nature of foodborne strains in fermented dairies, including several LAB strains of environmental origin with beneficial, although yet uncharacterised, features [28]. Their combined metabolic functions and metabolites have been suggested to exert positive effects on host physiology through synergistic mechanisms, more efficiently than single strain supplementation [43]. However, the probiotic capacity of mixed foodborne microbial consortia has been gaining consideration only recently [44–46]. Moreover, most published work report supplementation with single bacterial strains, and only few studies compared multi-strain probiotic mixtures to investigate possible synergistic interactions [47]. We chose to run a parallel group of mice for comparison, supplemented with the single probiotic strain GG of *Lactobacillus rhamnosus* that was shown to exert positive effects on obesity-related inflammation in mice and humans [31].

The obese phenotype was induced in C57BL/6J mice by feeding a 45% HFD for 3 months, resulting in weight gain in all experimental groups irrespective of bacterial supplementation type. Many other studies report decreased body weight gain following probiotic supplementation [31, 48]. Although we detected constant weight gain in all mice groups, decreased epididymal WAT weight was evident following oral administration of MBC microbiota as compared to the other mice groups, as well as a more pronounced anti-inflammatory effect than LGG supplementation. Decreased inflammation and amelioration of obesity-related metabolic and immunological dysfunctions were previously observed with bacterial supplementation of HFD-fed mice [49, 50], but they were not accompanied by WAT weight reduction. WAT is considered the main contributor to development of the obesity-associated low-grade chronic systemic inflammatory state, which is characterised by an imbalanced cytokine network with increased production of several pro-inflammatory mediators. Epididymal WAT, like other intra-abdominal WAT depots, is now recognised to have a more negative impact on health than subcutaneous WAT [51], and its decreased weight following MBC supplementation further highlights a higher efficacy of this complex microbial community in supporting healthy metabolism. The specific anti-inflammatory effects observed in our study involved decreased levels of the pro-inflammatory cytokines IL-6 and IFN-γ and of the chemokines IP-10 and RANTES in cultured WAT explants of LGG-supplemented mice, while MBC-treated animals displayed stronger decrease in the expression of a broader panel of pro-inflammatory cytokines and chemokines, namely IL-6, TNF-α, IL-17A, IFN-γ, IP-10, GM-CSF, and RANTES. Other studies using single probiotic strains or multi-strain mixtures observed decreased expression of some of these markers [48, 50, 52]. IL-6 and TNF-α are the main cytokines produced by pro-inflammatory macrophages in obese adipose tissue, whereas RANTES and IP-10 are important lymphocyte and macrophage chemo-attractants [9]. IFN-γ is secreted by infiltrating CD8^+^ T cells, thus contributing to the critical events driving adipose tissue inflammation [53]. Regarding IL-17, it was suggested that obesity predisposes to selective expansion of the Th17 subclass of T lymphocytes, producing high levels of IL-17 in an IL-6-dependent process [54]. The cytokine GM-CSF, although not frequently measured in studies addressing probiotic-dependent immunomodulation in obesity, was reported to increase in the serum of obese mice [55].

The positive effects exerted by MBC supplementation on the overall profile of WAT inflammatory cytokines and chemokines were also associated to improved balance between the major sub-populations of immune cells, as revealed by reduced percentage of pro-inflammatory CD8^+^ T lymphocytes, activated leukocytes and macrophages, and increased CD4^+^ T lymphocytes and CD25^+^Foxp3^+^ Treg cells. Similar findings were reported in other tissues following *Bifidobacterium pseudocatenulatum* supplementation [50], in the adipose tissue after *Lactobacillus gasseri* supplementation [56], or using a probiotic mixture of *L*. *rhamnosus* and *Bifidobacterium animalis* subsp. *lactis* [48]. Treg cells are highly represented in the WAT of lean mice, and they are essential for the maintenance of an anti-inflammatory environment in the absence of obesity. Treg cell number has been shown to decrease in the WAT of obese mice, contributing to worsen the inflammatory state [10, 11]. The increased Treg cell number that we observe after MBC supplementation is a result of particular relevance, considering that selective modulation of this population was shown to be tightly related to the level of obesity-associated inflammation [10].

The anti-inflammatory effects occurring with MBC supplementation were even more evident following PCA analysis of the datasets, which clearly discriminated MBC samples from LGG and CTRL samples along the first principal component axis. This confirms the key role of the immune cell subpopulations, as well as of the cytokines GM-CSF, IL-6, and TNF-α, as the most important variables contributing to the discrimination. Separation of the LGG and CTRL samples into two distinct clusters was highlighted only as a trend. These effects were accompanied by positive changes in the expression of lipid metabolism biomarkers in the MBC-supplemented group, with decreased circulating levels of triglycerides, increased HDL-cholesterol levels, and a trend toward decreasing LDL cholesterol. Higher levels of circulating HDL cholesterol were also observed in the LGG mice group, in line with previous reports on supplementation with single probiotics or mixtures [31, 48, 50].

Interaction with the host metagenome is considered an important aspect in probiotic-mediated immune stimulation [22, 57]. We analysed faecal microbiota biodiversity in treated mice by NGS of 16S rDNA. Our results confirmed that gut microbiota composition was indeed affected by HFD, leading to the establishment of an increased *Firmicutes*/*Bacteroidetes* ratio typical of the obesity pattern [58]. Bacterial supplementation was not able to overcome HFD-induced effects on gut microbial profile, as no substantial modifications in faecal microbiota composition could be observed over time by NGS. The overriding effect of HFD on microbial biodiversity was also confirmed by advanced multivariate statistical analysis, namely Principal Coordinates Analysis (PCoA), revealing no specific clustering of bacterial species according to supplementation type, while highlighting a clear variation of microbial composition at the end of the experimental period in all mice groups. Other studies reported different extent of alterations in resident gut microbiota profile following probiotic treatment of HFD-fed mice [48, 50, 59, 60], but the studies are not always comparable due to different experimental designs (duration of treatment, percent dietary fat, etc.) and experimental approaches employed for microbial profiling (i.e. NGS, qPCR). In our study, the high sensitivity of NGS allowed to detect two of the three predominant species characterising the MBC-derived microbiota, namely *L*. *delbrueckii* and *Leuc*. *lactis*, although with low relative abundance in the faecal microbiome of supplemented mice. These two species may thus be able to colonise the gut of supplemented mice more efficiently. Gut colonisation capacity of some components of MBC-derived microbiota was also shown in the simple model organism *Caenorhabditis elegans* [30]. On the other hand, the *L*. *rhamnosus* species that includes the LGG strain was undetectable in faecal microbiomes of LGG-treated mice. Conflicting results concerning LGG colonisation capacity have been reported in the literature. Park et al. recently observed decreased *Lactobacillus* relative abundances in the murine gut, including the LGG strain, following LAB supplementation [59], while in another report of orally administered LGG to knockout (ApoE−/−) mice fed HFD, *L*. *rhamnosus* could be recovered by faecal dilution and plating [61]. Nevertheless, several reports indicate that oral administration of specific bacteria can exert beneficial effects on the host even in the absence of colonisation [59, 62–64].

Taken together, our results suggest that supplementation with a biodiverse foodborne bacterial consortium can exert beneficial effects on obesity-associated inflammation and health-related parameters more effectively than single probiotic strain supplementation. A recent report by Sonnenburg et al. clearly shows that dietary perturbations can lead to permanent loss of specific gut bacterial taxa, due to negative selection of metabolic activities that become unnecessary under imbalanced dietary regimens [65]. These results point at limitations in microbiota resilience occurring under extreme conditions, such as HFD-induced obesity, where the alterations cannot be reversed by simple dietary intervention if not accompanied by specific bacterial supplementation aimed at restoring the lost taxa. Foodborne bacteria could play a key role in this respect, and to the best of our knowledge, this is among the very few reports evaluating the impact of a complex microbial consortium naturally occurring in a traditional fermented food on host physiology.

## Conclusions

Our results demonstrate a stronger effect of a mixed microbial consortium vs single-strain probiotic supplementation in ameliorating HFD-induced inflammation in the WAT of obese mice. The present study highlights the importance of considering complex foodborne microbial consortia naturally occurring in fermented products for human consumption as potential probiotic vectors. It also points at the importance of coupling multivariate to univariate statistical analysis for better understanding of the key factors responsible for probiotic effects. The observed immunomodulatory activity exerted by the MBC-derived microbiota suggests synergistic interactions of microbial strains of environmental origin, present within the foodborne consortium. More studies are needed to further investigate the role of dietary microbes with yet uncharacterised probiotic effect, aimed also at identifying novel, under-represented strains which could be unique to the foodborne microbiota.

## Additional file

Additional file 1: Table S1. Complete results of the identification, at the species level or, where not possible, at higher taxonomic ranks, of the sequences obtained by next-generation sequencing of faecal mice samples, expressed as percentage. Species representing the components of MBC microbiota are highlighted in bold. (XLSX 96 kb)

## Abbreviations

CFU: Colony-forming units
CTRL: Control
GM-CSF: Granulocyte macrophage-colony stimulating factor
HFD: High fat diet
IFN: Interferon
IL: Interleukin
IP: Interferon gamma-induced protein
LAB: Lactic acid bacteria
LGG: *L*. *rhamnosus* GG
MBC: Mozzarella di Bufala Campana
MRS: De Man Rogosa Sharpe medium
NGS: Next-generation sequencing
OTUs: Operational taxonomic units
PCA: Principal component analysis
PCoA: Principal Coordinates Analysis
RANTES: Regulated on Activation-Normal T cell Expressed and Secreted
TNF: Tumour necrosis factor
Treg: Regulatory T cells
WAT: White adipose tissue
